# Workpeople's Contributions to Hospitals: Threatened with Legislative Destruction

**Published:** 1914-02-28

**Authors:** 


### WORKPEOPLE'S CONTRIBUTIONS TO HOSPITALS.

### Threatened with Legislative Destruction.

When those responsible for providing the necessary income to maintain our voluntary hospitals were faced by a probable adverse effect upon the funds, through the passing of the National Insurance Act, they were particularly curious as to whether the working classes would consider that their contributions under the Health Act would, in some part, morally absolve them from supporting hospital funds. The question, put in other terms, practically was, How far do workmen regard their payments to hospitals in the light of club subscriptions? To gauge industrial feeling in the matter it was necessary to wait until January of last year, when the plan decided upon by the hospitals, in view of the commencement of the payment of benefits under the Act, was carried into effect. Then, in most places, insured hospital patients, whose ailments were such as can be treated by a competent medical practitioner, were referred to their panel doctors. The friction resulting from this action revealed the fact that there were many working people who actually did regard their contributions as qualifying them for medical and surgical treatment of all kinds at hospitals, should they so require, but it was pleasant to find that there was quite an equally large number who did not regard the contributions in the same light. The question was thrashed out at meetings of Hospital Saturday Funds and elsewhere, and in London an unsuccessful attempt was made to secure medical attention to all bearers of letters of recommendation from the Hospital Saturday Fund without further inquiry. As a result it was generally and finally made known that a contribution from a working man to a hospital does not free him from the application of the rules of the institution to his case, should he require treatment.

### Receipts from Hospital Saturday Funds.

When we come to examine the receipts from English Hospital Saturday Funds for 1911 and 1912, as shown in the new volume of "Burdett's Hospitals and Charities," we find that the totals for the two years are, respectively, £176,931 and £171,938. Of course, these large sums do not entirely come from the pockets of working people, including, as they do, grants from employers and other items. But being, as they must be, largely industrial collections, some idea of the effect of the Act in 1912 can be gathered therefrom; for during the last six months of 1912 the workman was paying a contribution to the State scheme as well as to the hospital. The comparison is interesting, but it is to the figures for 1912 and 1913 that we must look for the full effect of the legislation; the statistics are not yet all available, but in one or two places some totals may be quoted which give helpful data for use in reviewing the matter. In Nottingham, the total collections fell from £10,046 to £8,256, and from Sheffield, Manchester, Salford, Norwich, and Derby come reports of less important decreases. But, on the other hand, at Hull, Leicester, and Stoke-on-Trent increased support has been received. In London, the amount distributed in the two years by the Hospital Saturday Fund was as follows: 1912, £45,118; 1913, £40,309. As is stated in a circular issued by the Fund, dated January 13, 1914, "the perplexity caused by the operations of the National Insurance Act throughout the year undoubtedly accounted for the shrinkage." The London Hospital Saturday Fund executive are inclined to look upon the set-back as temporary, for in their case it can be demonstrated that it applied only to the first three quarters of the year, and perhaps this may also be the case in the provinces. Nevertheless, it says much for the working man and for the hospitals, who are proved to have retained his sympathy in so large a measure, that this important part of the revenue of our hospitals has not been more extensively affected.

### The New Truck Bill.

But it seems that another great test of the loyalty of working-class subscribers may have to be undergone. In July last a "Bill to amend and extend the Truck Acts, 1831 to 1896," sponsored by Lord Henry Cavendish Bentinck, Mr. Noel Buxton, Mr. O'Grady, Mr. Rowntree, and Mr. Stephen Walsh, was introduced into the House of Commons. Its object was to amend the Acts by making all deductions from wages and all fines illegal, and to extend the benefits (?) of the Acts to certain classes of workpeople not previously scheduled. The Bill touches the work of hospitals where it includes, amongst the things in respect of which an employer shall not make any deduction with or without the consent of an employee, "any subscription or payment to, or otherwise in connection with, any Benefit Society, Sick Club, Hospital, or any other society, club, or benefit, or for medical attendance." We are informed by the Bill Office of the House of Commons that the Bill quoted above was eventually dropped, but it is understood in political circles that legislation on the same lines is likely to be introduced at no far distant date. It will be remembered that the Truck Acts of 1831, 1887, and 1896, the amendment and extension of which are contemplated, were originally intended to rectify abuses by employers, whereby workpeople were made to pay exorbitant prices for goods supplied as part remuneration, or were subjected to fines of an unreasonable character. Certainly the spirit of these Acts has nothing in common with a movement to prevent workpeople from being allowed to give free consent to a convenient method of collecting willing contributions towards a local hospital.

As those who are familiar with hospital finance are well aware, the successful raising of funds depends largely upon the time and place chosen for collecting contributions. For instance, in one industrial centre the contributions from workmen yearly amount to between £18,000 and £19,000, and we are told that practically the whole of this sum is gathered in by the method of making a deduction, previously consented to, of a small sum from the workmen's wages. It is obvious that should the alternative method of receiving the few pence after the pay-window has been passed be resorted to, any amount of careful organisation would fail to get over the practical difficulties of collection. Indeed, in this instance alone, a competent authority estimates that the gross sum would be reduced by as much as one-half. As the workmen of the city in question have achieved the praiseworthy result of defraying half the ordinary expenditure of their local hospital, the feared outcome of the threatened legislation would be most disastrous to the sick poor of the neighbourhood.

Methods of Collection.

We have carefully inquired into the methods of collection in all parts of the country, and the probable effect of legislation; as the results are of general interest, some selected details may be set forth here. In London, it is found that the practice of making deductions from wages for any purpose whatever is rare, but the careful and widely extended operations of the Hospital Saturday Fund produce a goodly sum for the Metropolitan hospitals, chiefly as a result of industrial collections, although it may be observed that the total income of the Fund does not compare very favourably with the workmen's contributions, amounting to £18,693 in Newcastle-on-Tyne, where the population is roughly only a quarter of a million, and where there is no Hospital Saturday Fund. In Cardiff, King Edward VII.'s Hospital received £4,516 in 1913 from workmen's collections; the method of deduction is employed in the district, and an adverse effect is anticipated if the suggested amendment to the Acts is brought about. In Hull, the Royal Infirmary receives not far short of £4,000 from this source, 90 per cent. of which represents deduction, and a decrease of 50 per cent. is anticipated. The Hospital Saturday Society of Leicester contributed last year £15,632 to the Royal Infirmary. Here, as a rule, deductions are made by desire of the employees, and it is feared that the passing of the suggested Act will throw the institution back some ten years to the time when finances were raised by spasmodic efforts, necessitating much organisation with little result, and in all probability making a diminution in hospital activities. The same story comes from the infirmary at Stoke-on-Trent, whence the strong expression is made that if the proposed amendments to the Truck Act should pass, no power on earth can preserve the hospital as a voluntary institution.

In Leeds £14,000 yearly is collected in town and surrounding districts for the infirmary and other local charities. A large portion of this amount is deducted by consent from the wages of the men, and if the suggested alteration in the Truck Acts were made, it would doubtless affect the General Infirmary very considerably. In Norwich, where the amount is not actually deducted from the employees' wages, they are given to understand that it is the custom in the factories for each workman to give one penny monthly. In Derby the system of deduction is prevalent. Most collections go through the hands of the Hospital Saturday Fund, and it is thought that the Fund would cease to exist in the district if this method of collection were done away with. In none of these centres does one find evidence of any irritation felt by the workpeople because of the methods used in collection.

Beware of Uncalled-for Legislation.

Under the circumstances it is not surprising to learn that in spite of the fact that the proposed legislation has for a time been put on one side, the matter continues to receive the earnest consideration of the hospital executive bodies. The hospitals and the working man have come through the troubles and trials of the early operations of the National Insurance Act without seriously damaging their friendly relationship; let us now hope that this further and uncalled-for interference with their ancient alliance shall be permitted to continue to repose in the limbo of unasked-for proposals.

